# Hallucination: A key challenge to Artificial Intelligence-Generated writing

**DOI:** 10.51866/lte.527

**Published:** 2023-11-28

**Authors:** Jazlan Jamaluddin, Nadia Abd Gaffar, Nor Shazatul Salwana Din

**Affiliations:** 1 MD, MMed, Klinik Kesihatan Sauk, Jalan Besar Lenggong, Sauk, Kuala Kangsar, Perak, Malaysia. Email: jazlanjamaluddin@gmail.com; 2 MD, MMed, Klinik Kesihatan Tanjung Malim Jalan Besar, Tanjung Malim, Perak, Malaysia.; 3 MD, MMed, Klinik Kesihatan Bota Kiri, Jalan Bota Kiri, Bota Kiri, Perak Tengah, Perak, Malaysia.

**Keywords:** Artificial intelligence, Hallucinations, Delusions, Medical writing, Biomedical research

## Dear editor,

We read the article by Lee et al. with great interest.^1^ Artificial intelligence (AI) has become an indispensable tool not only in academic writing, particularly among students, trainees and other related individuals, but also in clinical and clerical work, especially in resource-limited and rural clinics, where it continues to aid clinicians in drafting letters, memos, dossiers and other documents. With the increasing use of AI, including but not limited to systems such as ChatGPT, in various facets of daily life, it is crucial to address the issue of ‘hallucination’ in AIgenerated content. This issue has been gaining prominence in the field of AI this year after the launch of ChatGPT in late November 2022.^2^

In AI, hallucination (also known as artificial hallucination or confabulation) occurs when responses given are not factually accurate or do not correspond to any real-world data.^2,3^ It arises when a generative model is asked to produce text, image or other content without strict constraints or clear guidance. Generative models are trained on vast amounts of data and learn to generate content based on patterns they have observed in such data. When they generate content without specific guidelines, they may draw upon unusual or unexpected patterns they have learnt, leading to outputs that seem strange, surreal or disconnected from reality. This issue can occur owing to errors in AI systems’ training data, overfitting of AI systems and a lack of understanding of the underlying biomedical concepts. This becomes especially problematic when citing references or sources that are non-existent or inaccurate. While AI tools can be used for citation and reference management, many of the references generated are often fabricated.^4^ When using AI-generated content, authors need to exercise vigilance when AI references external sources. The output provided by AI often depends on the prompts given by its users, the version of model used and its ability to access the internet. It is not enough to simply rely on AI’s ability to generate citations. Authors should take the time to find and read through the referenced articles, ensuring their accuracy and relevance. This additional step is essential to maintain the integrity of the academic and professional work produced with the assistance of AI. Unfortunately, there have been increasing instances of authors simply referencing articles without reading them or, worse, referencing non-existent articles. This phenomenon has been reported worldwide and poses a significant challenge to the credibility of scholarly publications and even official documentation.^3,5,6^

As Lee et al. are part of the editorial team of this esteemed journal, it is important to also emphasise the proactive role of editors and reviewers in addressing this issue.^2^ When evaluating manuscripts submitted to the journal, editors and reviewers must double-check not only the content but also the references provided. The easiest way to confirm the validity of a reference is by visiting the webpage link provided. In addition to manual verification, there are various software tools available that can help confirm whether AI has been used in the writing of an article, most of which have been included in plagiarism detection software. These tools can assist in identifying potential instances of hallucinations in AI-generated content. Such software can be valuable in the fight against misinformation and inaccuracies in academic and professional discourses.

Just as hallucinations in human cognition are acknowledged and addressed as part of World Mental Health Day every year on October 10, it is equally important to recognise and address the phenomenon of hallucinations in AI-generated content. Notably, AI hallucinations are not actual hallucinations in the psychological sense, as they are not experienced by AI tools themselves but rather a result of models’ pattern recognition and generation processes. Researchers and developers are continually working to improve AI systems and reduce the occurrence of such hallucinatory outputs while making them more useful and aligned with human intent. However, managing hallucinations in AI is akin to managing cognitive distortions in human thinking. Authors must take the responsibility to corroborate the facts provided by AI and ensure the accuracy of their references. As the use of AI continues to grow in various aspects of life, including academia and professional work, it is imperative to address the issue of hallucinations in Al-generated content. In the realm of clinical papers and research, although there exists a genuine requirement for a validated AI tool designed specifically for referencing and citation purposes, authors, editors and reviewers all have roles to play in mitigating this problem and upholding the standards of accuracy and integrity in publications. Human touch remains important when using AI, and AI-generated content should be used with caution. While advancements in AI hold promise for increased reliability, complete replacement of humans by AI remains improbable at this point in time.
